# Alteration in coracohumeral ligament and distance in people with symptoms of subcoracoid impingement

**DOI:** 10.1186/s12891-023-06152-z

**Published:** 2023-01-23

**Authors:** Deng Siang Ting, Jing-lan Yang, Kwan-Hwa Lin, Tyng-Guey Wang, Jiu-Jenq Lin

**Affiliations:** 1 School and Graduate Institute of Physical Therapy, College of Medicine, National Taiwan University, Taipei City, Taiwan; 2 Division of Physical Therapy, Department of Physical Medicine and Rehabilitation, National Taiwan University Hospital, Taipei City, Taiwan

**Keywords:** Coracohumeral distance, Coracohumeral ligament, Subcoracoid impingement, Subscapularis

## Abstract

**Background:**

Differentiation between subacromial impingement versus subcoracoid impingement are important for the treatment target. We evaluated the correlations between coracohumeral ligament (CHL) thickness and distance (CHD) and characterized the CHL and subscapularis (SSC) in subcoracoid impingement subjects.

**Methods:**

An observational, cross-sectional study was carried out. Twenty subcoracoid impingement subjects and age/gender matched controls were assessed in 4 different shoulder positions by ultrasonography.

**Results:**

Moderate correlations between CHL thickness with CHD (*r* = 0.455 in neutral rotation, *p* = 0.044; *r* = 0.483 in interior rotation, *p* = 0.031) were found in subacromial subjects. Subcoracoid impingement subjects had greater CHL thickness (difference = 0.3 mm, effect size = 0.85, *p* = 0.006), SSC tendon thickness (difference = 0.7 mm, effect size = 0.92, *p* = 0.01) and SSC/CHD occupation ratio (difference = 8%, effect size = 0.95, *p* = 0.005) compared with the control.

**Conclusions:**

Coracohumeral distance is related to ligament thickness, especially in subacromial impingement subjects. Increased coracohumeral ligament and subscapularis thickness as well as decreased subscapularis/coracohumeral distance occupation ratio are characterized in subcoracoid impingement subjects. These quantitative measurements can be useful in identifying patients at risk of subcoracoid impingement from subacromial impingement.

**Supplementary Information:**

The online version contains supplementary material available at 10.1186/s12891-023-06152-z.

## Background

Shoulder impingement syndrome (SIS) is a broad term that is often used to label indefinite pain around the shoulder. Although SIS usually refers to subacromial impingement, the literature on anterior impingement also describes subcoracoid impingement [1]. While subacromial impingement is defined as inflammation and irritation of the rotator cuff tendons as they pass through the subacromial space, subcoracoid impingement is defined as entrapment of the subscapularis within the subcoracoid space between the posterolateral coracoid process and the lesser tubercle of the humerus [2]. Despite subcoracoid impingement being relatively uncommon compared to subacromial impingement, 19% of patients with rotator cuff tears have both subacromial and subcoracoid impingement [3]. Untreated subcoracoid impingement is the cause of constant anterior shoulder pain in 5% of patients with rotator cuff repairs [3]. Prevalence of subcoracoid impingement of 13%–56% has also been reported in patients with subscapularis tears [4, 5]. Subcoracoid impingement is the focus of this investigation.

Subcoracoid space filling and narrowing are proposed as the underlying causes of subcoracoid impingement. Anterosuperior migration of the humeral head in rotator cuff deficient shoulders and anatomic variation of the coracoid/lesser tuberosity/bicipital groove have been documented [6, 7]. The acromiohumeral and coracohumeral spaces can be quantified by using ultrasound to measure the distance between the inferior edge of the acromion and the superior part of the humeral head (the acromiohumeral distance, AHD) as well as that between the posterolateral coracoid process and the lesser tubercle of the humerus (the coracohumeral distance, CHD) [8]. A CHD of less than 6 mm can eventually result in subscapularis tendon failure. Both the AHD and CHD respond to the position of the humerus and are related to the clinical symptoms of SIS [8–10].

The function of the subscapularis (SSC) muscle should be considered as one of the important factors in subcoracoid impingement [2]. SSC pathology develops in around 14% of shoulders with posterosuperior rotator cuff tear. Subjects with SIS have high prevalence (71%) of subscapularis-torn shoulders [10, 11]. Thus, the differential diagnosis of subacromial impingement and subcoracoid impingement has clinical importance.

The CHL has been indicated as an inferior stabilizer and external rotation restraint of the humerus, which are related to shoulder instability and stiffness, respectively [12, 13]. Proximally, the CHL attaches from the lateral aspect of the base of the coracoid process. Distally, it attaches onto 2 parts, which are the posterior portion of the greater tubercle and the cranial part of the subscapularis muscle. It has been suggested to be a sustainer of the subscapularis, protecting the subscapularis by enveloping its tendon in the shoulder abduction and exterior rotation positions [14]. Histologically, the CHL is not a true “ligament” but has a similar appearance to a shoulder capsule with loose connective tissue [14]. From these anatomic and histological aspects of the CHL, we hypothesize that patients with SIS can show significant negative correlation between CHL thickness and CHD on the affected side with and without SSC lesion. We also hypothesize that SIS patients with SSC lesion can have significantly increased thickness in the CHL and decreased CHD on the affected side compared to those of SIS patients without SSC lesion. The purposes of the study were (1) to evaluate the correlations between the CHL thickness and CHD by using ultrasonography in people with SIS with and without SSC lesion, and (2) to examine the differences in SSC/CHL thickness and CHD between people with SIS with and without SSC lesion.

## Method

This cross-sectional observational study is a level 3 study. The thickness of CHL and CHD, measured by USG, were compared between SIS patients with and without SSC lesion. This study was approved by University Hospital Ethical Review Board for Medical Research Involving Human Subjects, and each participant gave his/her informed consent.

### Subjects

Based on previous studies, a total sample size of 40 participants was calculated to provide 80% power with detection of a difference of more than 1.0 mm CHD between 2 groups [8]. The inclusion criteria of the participants were age of 20–60 years old and positive unilateral shoulder results on at least 3 of the 5 tests: (1) Neer's test, (2) Hawkins' test, (3) the Empty can test, (4) the pain or weakness with resisted ER test, and (5) tenderness in the tendon of the rotator cuff [15, 16]. Participants with a history of shoulder dislocation, fracture or surgery, history of direct contact injury to the neck or upper extremities within the past month, glenohumeral joint instability (positive apprehension test, sulcus sign), neurologic disorder (upper motor neuron diseases, cervical radiculopathy), passive ER ROM < 30°, or pain (visual analogue scale, VAS > 5) during the experimental tasks were excluded. After the impingement tests were performed to ensure that the participants met our inclusion criteria, all participants were assessed with 3 special SSC tests, namely, (1) the lift-off test, (2) belly-press test (Napoleon sign) and 3) bear-hug test [17, 18] for group allocation. Patients with positive results on at least 2 of the 3 special tests were allocated to the SSC lesion (SSCL) group. SSC tears and cuff lag signs were also excluded as they may falsely affect the CHD due to resting anterior positioning of the humerus. To clarify SSC lesion without tears, integrity of the biceps pulley was confirmed by USG.

### Instrumentation and procedures

The T3300 ultrasound system (BenQ, Taipei, Taiwan), a portable ultrasonography machine, was used to assess the thickness of the CHL, SSC tendon, and supraspinatus (SSP) tendon, as well as the CHD and acromiohumeral distance (AHD). A L154BH linear array probe with a range of 4–15 MHz was used to collect data. A B-mode USG with preset musculoskeletal (MSK) examining parameters (gain, 64; dynamic range, 75; QScan, 4; persistence, 3; gray map, 3; chroma map, 0; and steering angle, 0) was used to collect data. Depending on the depth of the structure we wanted to measure, tissue harmonic imaging (THI) was turned on at 5.5 MHz for better resolution and turned off at 12 MHz for better penetration. Previous studies have shown relatively higher accuracy and reliability in measurement of shoulder pathologies [19, 20].

**Fig. 1:**
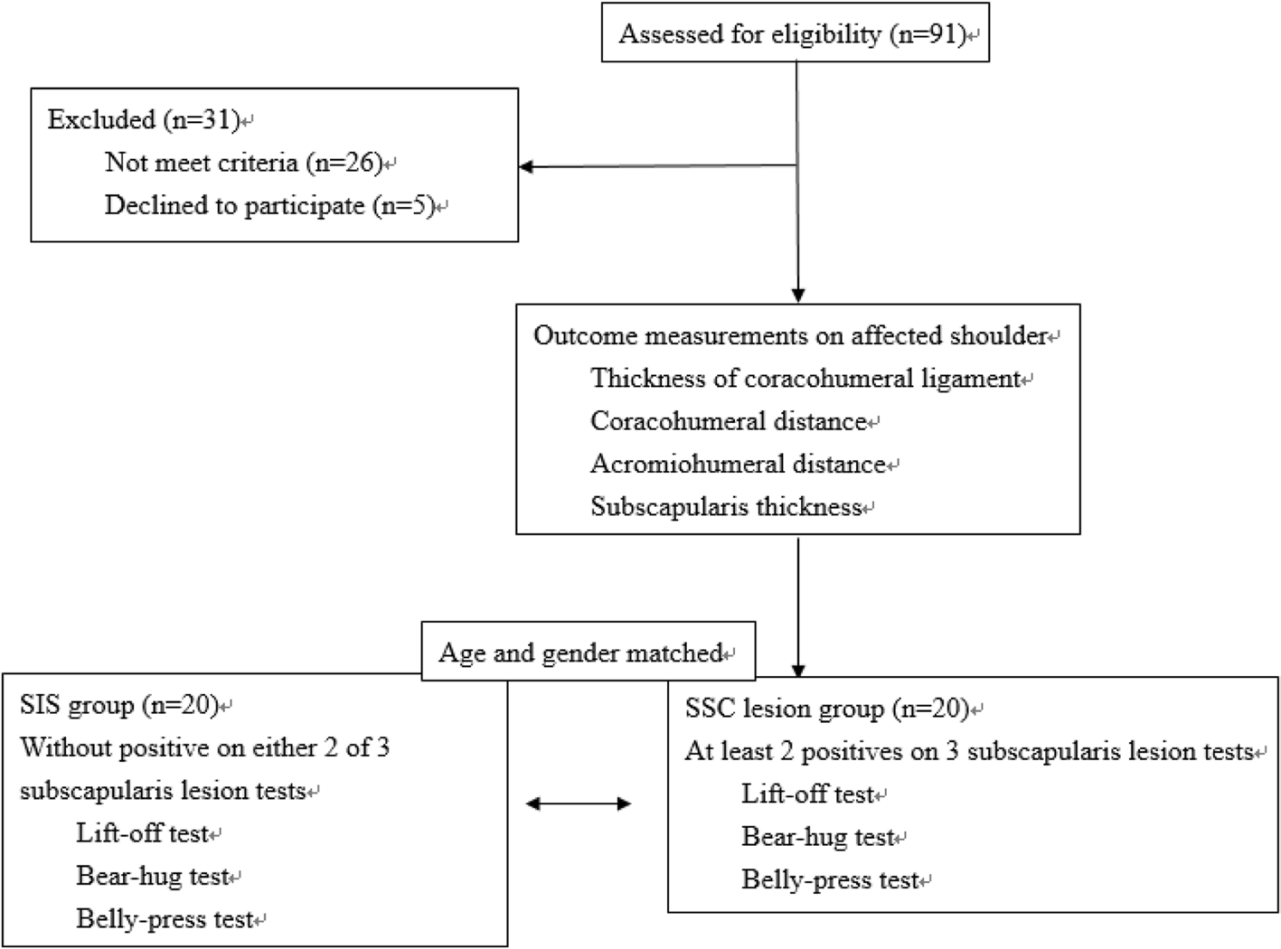
Flowchart of the experiment

The experimental procedure is illustrated in Fig. 1. The characteristics of the participants were collected by one assessor, including age, gender, height, weight, dominant side, involved side, duration of symptom, pain (VAS), occupation ratio, Flexilevel scale [21] and internal rotator strength.

### USG measurements

We measured the following outcomes: (1) CHL thickness, (2) CHD, (3) SSP thickness, (4) SSC thickness and (5) AHD. Each outcome was measured in 3 trials and the mean of the 3 trials was used for data analyses. All of the measurements were measured with THI turned on except for that of CHD, due to the deeper anatomical structure.

**Fig. 2:**
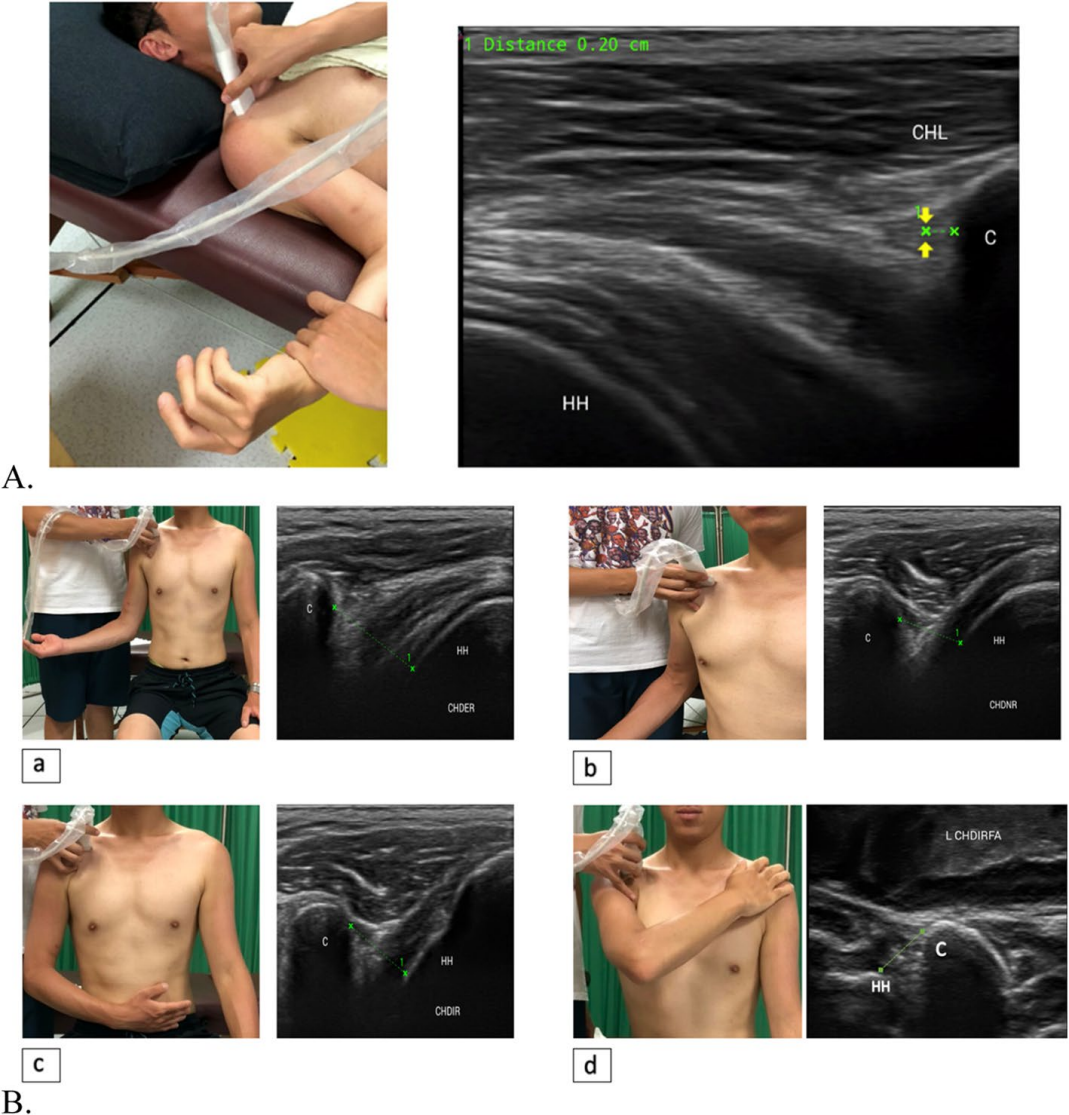
The illustration of USG measurement. A. coracohumeral ligament (CHL) B. a. coracohumeral distance (CHD) in external rotation (ER), b. neutral rotation (NR), c. internal rotation (IR) and d. internal rotation with maximal flexion and adduction (IRFA). CP: coracoid process HH: humerus head, C: coracoid process HH: humerus head

For measurement of the CHL thickness, the position of the linear probe was on the lateral border of the coracoid process to obtain a longitudinal image of the CHL. Each participant was instructed to lie in supine position and relax while the examiner maintained the elbow of the participant at flexion of 90° and the shoulder under maximal ER without shoulder abduction or flexion (arm by side) (Fig. 2). Maximal ER of the shoulder was achieved when the examiner could not further externally rotate the shoulder of the participant. The thickness of the CHL at a 2-mm distance from the coracoid process was measured. The intraclass correlation coefficient (ICC) of CHL thickness was 0.854 with 0.2 mm standard error of measurement (SEM).

CHD was measured with the probe positioned on the lateral border of the coracoid process to obtain images of the coracoid process and humeral head in 4 different shoulder rotation positions: (1) shoulder neutral rotation (CHD-NR), (2) external rotation (CHD-ER) and (3) shoulder internal rotation with maximal forward flexion and full adduction (CHD-IRFA, with the arm adducted across the chest reaching for the opposite shoulder) and (4) shoulder internal rotation (CHD-IR) (Fig. 2) [8, 20]. Participants were asked to sit with their arms by their sides and to perform the 4 different positions respectively. The measurements were repeated for 3 trials with repositioning of the arm to a neutral position for intervals of 10 s. The distance measured was that between the coracoid process and the lesser tuberosity of the humerus. The ICCs of CHD were 0.996, 0.969, 0.893 and 0.930 with 0.1 mm, 0.3 mm, 0.5 mm and 0.4 mm SEMs respectively.

SSP tendon thickness was evaluated with the patient's palm placed over his/her iliac wing, or “back pocket”, with the elbow flexed and directed medially [22]. The transducer was placed over the anterior aspect of the shoulder, perpendicular to the supraspinatus tendon and just anterior of the anterior–lateral margin of the acromion. A transverse glide was then performed at the site to determine the exact position where the observer judged that the tendon thickness was at its maximum. The thickness of the SSP tendon was measured 2 cm away from the biceps long head tendon [23]. For measuring SSC tendon thickness with a short axis of view, the probe was positioned horizontally on the bicipital grove. The participant’s forearm was placed with the elbow flexed to 90° in slight internal rotation, with the palm facing upward and medially. Then the patient was asked to rotate the forearm externally, keeping the palm up and the elbow strictly close to the iliac crest. The ICC of SSP tendon thickness was 0.943 with 0.2 mm SEM.

AHD was measured under 0° and 60° of scapular plane shoulder elevation. The transducer was placed on the most anterior aspect of the acromion edge, with the long axis of the transducer placed in the plane of the scapula and parallel to the flat surface of the acromion [22]. The participants sat in an upright position without back support and with their feet flat on the floor, holding their shoulders back and looking straight ahead, to achieve retracted shoulders and extension in the thoracic and cervical spine. AHD was measured at 0° and 60° of active shoulder elevation in the scapular plane. A 60° scapular elevation of AHD was then measured with a goniometer placed on the patient's arm to determine 60° of active shoulder elevation, with the thumb pointing up. The ICCs of AHD were 0.963 and 0.982 with 0.1 mm and 0.1 mm SEMs respectively.

### Statistical analysis

All data were analyzed by the assessor who performed the USG measurement in SPSS 25.0 (IBM, USA), with an α level 0.05. The Shapiro–Wilk test was used for the test of normality. Demographic data were calculated by independent t-test for normally distributed data or Mann–Whitney U test for non-normally distributed data. Correlation of CHL and CHD was calculated by Pearson correlation coefficient for normally distributed data or Spearman correlation coefficient for non-normally distributed data. Group differences in CHL and CHD were tested by independent t-test for normally distributed data or Mann–Whitney U test for non-normally distributed data.

## Results

**Fig. 3:**
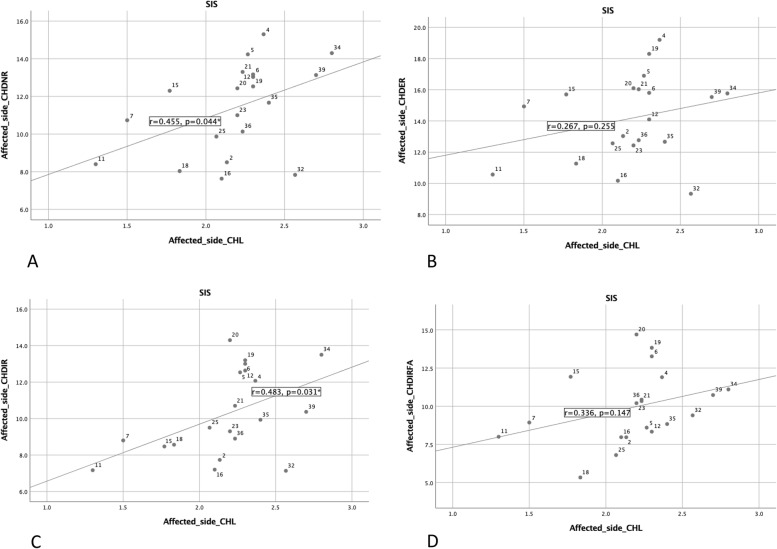
Correlation between CHL thickness and CHD on affected side of SIS. A: CHD-neutral rotation (NR), B: CHD-external rotation (ER), C: CHD-internal rotation (IR), D: CHD-internal rotation + maximal degrees flexion + adduction (IRFA). SIS: shoulder impingement syndrome group, SSCL: subscapularis lesion group, CHL: coracohumeral ligament, CHD: coracohumeral distance, SSP: supraspinatus, SSC: subscapularis, AHD: acromiohumeral distance

Ninety-one subjects were recruited for the study; 26 did not meet the criteria and 5 declined. Twenty subjects were met the criteria as SSC lesion while age and gender-matched 20 subjects were allocated as SIS group (Table 1). The outcome measurements of USG are shown in Table 2. The correlation between CHL thickness and CHD is shown in Fig. 3. In the affected side of the SIS group, moderate correlations between CHL thickness and CHD (CHDNR: *r* = 0.455, *p* = 0.044; CHDIR: *r* = 0.483, *p* = 0.031) were found. There was no other significant correlation between CHD and CHL variables.

**Table 1:** Demographic data of the subjects

Demographic data	SIS (*n* = 20)	SSCL (*n* = 20)
Age (year)	27.3 ± 6.8 (25.2)	27.5 ± 7.8 (26.0)
Gender (Male:Female)	7:13	7:13
Height (cm)	167.0 ± 2.4 (166.0)	168.0 ± 1.8 (166.5)
Weight (kg)	62.2 ± 8.1 (63.5)	65.4 ± 16.9 (60.0)
Dominant arm (Right:Left)	19:1	18:2
Affected arm (Right:Left)	17:3	14:6
Duration (month)	16.0 ± 16.1 (12.0)	21.0 ± 30.2 (6.5)
Visual analog scale	5.4 ± 0.4 (6.0)	5.2 ± 0.4 (5.0)
Flexilevel Scale	35.6 ± 4.7 (36.0)	35.4 ± 4.6 (35.0)

*SIS* shoulder impingement syndrome group, *SSCL* subscapularis lesion group mean ± standard deviation (median)

**Table 2:** Primary USG Measurement

USG outcomes		SIS (*n* = 20)		SSCL (*n* = 20)	
		Affected side	Non-affected side	Affected side	Non-affected side
CHL thickness (mm)		2.2 ± 0.4 (2.2)^*,#^	1.6 ± 0.2 (1.6)^*,#^	2.5 ± 0.3 (2.4)^*,#^	1.8 ± 0.3 (1.7)^*,#^
CHD (mm)	Neutral Rotation	11.4 ± 2.4 (12.0)	11.5 ± 2.5 (11.8)	10.3 ± 2.3 (10.3)	10.8 ± 2.2 (11.8)
	External Rotation	14.2 ± 2.7 (14.5)	14.5 ± 2.7 (15.0)	13.2 ± 2.5 (13.4)	13.6 ± 2.0 (13.5)
	Internal Rotation	10.3 ± 2.3 (9.7)	10.8 ± 2.2 (11.0)	9.4 ± 2.6 (9.8)	10.2 ± 2.0 (9.5)
	IRFA	9.9 ± 2.4 (9.8)	11.0 ± 1.8 (11.2) ^*^	8.7 ± 2.3(8.8)	9.6 ± 1.7 (9.7) ^*^
AHD (mm)	0°	8.9 ± 1.3 (8.6)	8.8 ± 1.6 (8.6)	9.4 ± 1.9 (8.8)	9.0 ± 1.3 (9.2)
	60°	5.3 ± 1.1 (5.1)	5.5 ± 1.4 (5.1)	5.5 ± 1.4 (5.1)	5.5 ± 1.0 (5.5)
Tendon thickness (mm)	SSP	5.9 ± 0.9 (6.0)	5.4 ± 0.6 (5.4)	6.4 ± 1.2 (6.3)	5.7 ± 1.0 (5.3)
	SSC	4.9 ± 0.6 (4.9)^*^	5.0 ± 0.7 (5.0)	5.6 ± 0.9 (5.4)^*^	5.3 ± 0.8 (5.2)
Occupation ratio (%)	SSP/AHD	67.4 ± 12.1 (67.5)	63.7 ± 14.9 (59.0)	68.7 ± 12.4 (67.5)	63.4 ± 9.8 (63.5)
	SSC/CHDNR	45.0 ± 11.0 (42.0)^*^	45.2 ± 12.8 (41.0)	56.2 ± 12.5 (54.0)^*^	50.6 ± 10.2 (52.0)

*SIS* shoulder impingement syndrome group, *SSCL* subscapularis lesion group

mean ± standard deviation (median), *USG* ultrasonography, *CHL* coracohumeral ligament, CHD-coracohumeral distance, *IRFA* internal rotation + 90° forward flexion + adduction, *LHB* long head of biceps, *AHD* acromiohumeral distance, *SSP* supraspinatus, *SSC* subscapularis, *CHDNR* coracohumeral distance in neutral rotation

^*^significance difference between the 2 groups, *p* < 0.05

^#^significance difference between affected side and non-affected side, *p* < 0.0001

The CHL thicknesses of the two sides showed significant differences between the two groups (affected side: difference = 0.3 mm, effect size = 0.85, *p* = 0.006; non-affected side: difference = 0.1 mm, effect size = 0.70, *p* = 0.034). The CHD in 4 positions of the affected and non-affected sides showed no significant differences between the two groups, except for the non-affected side CHDIRFA (difference 1.4 mm, effect size = 0.82, *p* = 0.004). Additionally, the CHL thickness was significantly different between the affected and non-affected sides respectively in the two groups (SIS: difference = 0.6 mm, effect size = 1.08, *p* < 0.0001; SSCL: difference = 0.7 mm, effect size = 2.33, *p* < 0.0001). There were also significant differences between the 2 groups in SSC tendon thickness on the affected side (difference = 0.7 mm, effect size = 0.92, *p* = 0.01) and occupation ratio of SSC/CHDNR (difference = 8%, effect size = 0.95, *p* = 0.005).

## Discussion

Although subcoracoid impingement is relatively uncommon compared to subacromial impingement (SIS) [3], it has been proposed as one of the causes of anterior shoulder pain in patients [1–3]. For distinguishing subacromial impingement from subcoracoid impingement, we hypothesized that the thickness of CHL would affect CHD in SIS patients with subscapularis lesion. This study investigated the influences of SSC lesion in SIS patients by USG measurement.

It is believed that repetitive impingement of soft tissue would cause micro-trauma of the surrounding tissues, which might further result in thickening of the collagen fibers and possible impingement [24, 25]. The difference in CHL thickness between the two groups might indicate different alteration patterns of soft tissue after impingement. SIS patients with SSC lesion had more CHL thickening than did SIS patients. Compared to the unaffected side, the thickening of the CHL on the affected side was similar in both groups (difference: SIS = 0.6 mm and SSCL = 0.7 mm). Previous studies also reported thicker CHLs in other shoulder pathologies, such as frozen shoulder and anterior instability [26, 27]. Thus, the CHL thickness in SIS patients could be related to the occurrence of subcoracoid impingement.

The measurement of CHD in different positions might be a feasible method of investigating potential subcoracoid impingement. Measuring CHD in 3 shoulder positions, Oh et al. [8] found narrowed CHD in the IRFA position in SSC tear patients. Similar to this finding, our results showed that the CHD was narrowest in IRFA and widest in ER positions. Reichel et al. [26] suggest that a CHD width of less than 9.5 mm can predict SSC lesion. Regarding our results, the narrow CHD in CHDIR (9.4 mm) and CHDIRFA (8.7 mm) on the affected side might cause possible impingement and subsequently SSC lesion. CHD measurement in the IR or IRFA positions can be used to characterize SIS patients with SSC lesion.

Both the subacromial and subcoracoid spaces can be characterized by AHD and CHD respectively, which would help shed light on the underlying mechanism of impingement. Michener et al. [28] found significant increases in the supraspinatus (SSP) tendon thickness and occupation ratio of SSP/AHD in SIS patients. Similar to this occupation theory, our results showed increased SSC tendon thickness and occupation ratio of SSC/CHDNR in SIS patients with SSC lesion. A greater SSC tendon thickness can occupy more subcoracoid space (SSC/CHDNR) and induce subcoracoid impingement and SSC lesion. To prevent narrow occupation in the subacromial and subcoracoid spaces, treatment strategies such as stretching or massage on the SSP and SSC are suggested for reducing tendon thickness and occupation of the related space.

The occupation ratio is an important factor for characterization of SSC lesion. In our results, the SSCL group showed significant increases in the occupation ratio of SSC/CHDNR on the affected side. Researchers also found a significantly smaller CHD in patients with SSC tear [8, 9]. However, Tollemar et al. [29] found no significant differences in CHD between patients with and without SSC lesion. The different results might have been due to the variation of severity and sample size of patients with SSC tear recruited. Tollemar et al. [29] reported that 10.1% of patients with rotator cuff tear had complete SSC tear. In addition, Cigolotti et al. [30] reported that over 50% of rotator cuff tears were age-related and that a surgical approach to repair SSC tears was essential to the outcome. We believed that early detection of SSC injury could reduce the future need for a surgical procedure. In the current study, no obvious signs of SSC tear were observed in the SSCL group. Since we found significant differences between the two groups only in CHL thickness and occupation ratio and not in CHD, evaluation of CHL thickness and occupation ratio of SSC/CHDNR can be included in the differential diagnosis of SIS patients with and without SSC lesion.

The inconsistent alteration of increasing CHL thickness and CHD might be a factor leading to SSC lesion. In contrast to the significant negative correlation between CHL thickness and CHD on the affected side in the patients with SIS that we had hypothesized, we found moderate positive correlation on the affected side in the SIS group. The different trend of correlation in the SSCL group might be an indicator of different alteration patterns of CHD with respect to CHL thickening. Thus, increased CHL with decreased CHD would worsen the impingement. The assessment of CHL thickness, CHD and SSC tendon thickness could be conducted in SIS patients to characterize possible further SSC lesion.

The limitations of current study should be noted. First, the experimental setting did not simulate actual situations, for both measurements were conducted at specific positions. This approach might not be able to induce the impingement that occurs during functional movements. Second, the cross-sectional study design might not able to explain the whole scenario of SIS patients with SSC lesion. Lastly, a healthy control group can be recruited to differentiate the outcomes of USG measurements for further clinical reference.

## Conclusions

This study evaluated the correlations between the CHL thickness and CHD by using USG in people with SIS with and without SSC lesion and examined the differences in SSC/CHL thickness and CHD between people with SIS with and without SSC lesion. CHL thickness plays an important role in CHD, especially in SIS patients without SSC lesion. Evaluation of CHL thickness, CHD and occupation ratio of SSC/CHDNR can be used to characterize SIS patients with SSC lesion. Additionally, the inconsistent alteration of increasing CHL thickness and CHD might be a factor in the development of SSC lesion in SIS patients. These quantitative measurements can be useful in identifying patients at risk of subcoracoid impingement from subacromial impingement.

## Supplementary Information

**Additional file 1.**

## Data Availability

The datasets used and/or analyzed during the current study are available from the corresponding author on reasonable request. They are not publicly available as they are currently part of a prospective cohort that will be used for future analyses.
