# Supplementary material for: Alteration in coracohumeral ligament and distance in people with symptoms of subcoracoid impingement
Source: BMC Musculoskelet Disord. 2023 Jan 23;24:58. doi: 10.1186/s12891-023-06152-z (PMC9869551; doi:10.1186/s12891-023-06152-z)
Supplement: Supplementary file 1 — Additional file 1. [file 12891_2023_6152_MOESM1_ESM.pdf]

正本

國立臺灣大學醫學院附設醫院　函

地　址：臺北市中山南路7號
聯絡人：王劭慈
電　話：(02)23123456　分機　63157

受文者：國立臺灣大學醫學院物理治療學系暨研究所林居正教授
發文日期：中華民國109年12月9日
發文字號：校附醫倫字第1093706522號
速別：普通件
密等及解密條件或保密期限：
附件：招募文宣202011045RIND

主旨：有關台端所主持之「肩胛下肌損傷於肩夾擠症候群患者之影響：超音波影像與肩胛骨運動學研究/The Influences of Subscapularis Lesion on Ultrasonography and Scapular Kinematics in Patients with Shoulder Impingement Syndrome」（本院案號：202011045RIND）學術臨床試驗/研究案，符合簡易審查條件及研究倫理規範，通過本院D研究倫理委員會審查，同意核備，並提第109次會議報備追認，請查照。

說明：

一、本臨床試驗/研究核准之有效期限自發文日起1年，計畫主持人應於許可到期日前10週至前6週向研究倫理委員會提出持續審查申請，並經審查同意後，方可繼續執行，且於填報持續審查/結案報告前須先至研究倫理委員會PTMS系統登錄第一位個案收案時間。

二、本臨床試驗/研究計畫若需變更、暫停執行、中途終止或結束時，主持人應向本會提出審查申請。計畫主持人並須依國內相關法令及本院規定通報嚴重不良反應事件及非預期問題。

三、本院研究倫理委員會同意之文件版本日期如下：

（一）臨床試驗/研究計畫書：V3. 2020/12/03。

（二）中文摘要：V1. 2020/11/10。

（三）受試者說明及同意書：V3. 2020/11/27。

ID: 202011045RIND The Influences of Subscapularis Lesion on Ultrasonography and Kinematics in Patients With Shoulder Impingement NCT05371457

Record Summary

Home Help

Record Status

In Progress → Entry Completed → Approved → Released → PRS Review → *Public*

Reset to In-Progress...

Record Owner:	NTUH	Access List:	[] Edit
Last Update:	05/11/2022 22:03 by NTUH	Upload:	Allowed Edit
Initial Release:	02/06/2022	PRS Review:	Review History
Last Release:	05/11/2022 Receipt (PDF)	Public Site:	Last Public Release: 05/11/2022 View on ClinicalTrials.gov
		FDAAA:	Non-ACT (Not interventional)

Spelling Preview Draft Receipt (PDF RTF) Download XML

Open

Protocol Section

Identifiers: NCT05371457 Unique Protocol ID: 202011045RIND

Brief Title: The Influences of Subscapularis Lesion on Ultrasonography and Kinematics in Patients With Shoulder Impingement

Module Status:

- Study Identification: ✔
- Study Status: ✔
- Sponsor/Collaborators: ✔
- Oversight: ✔
- Study Description: ✔
- Conditions: ✔
- Study Design: ✔
- Groups and Interventions:
- Outcome Measures: ✔
- Eligibility: ✔ 1 Note
- Contacts/Locations: ✔
- IPD Sharing Statement:
- References:
